# Mechanical and Abrasive Wear Performance of Titanium Di-Oxide Filled Woven Glass Fibre Reinforced Polymer Composites by Using Taguchi and EDAS Approach

**DOI:** 10.3390/ma14185257

**Published:** 2021-09-13

**Authors:** Chelliah Anand Chairman, Manickam Ravichandran, Vinayagam Mohanavel, Thanikodi Sathish, Ahmad Rashedi, Ibrahim M. Alarifi, Irfan Anjum Badruddin, Ali E. Anqi, Asif Afzal

**Affiliations:** 1 Department of Mechanical Engineering, K. Ramakrishnan College of Engineering, Trichy 621112, India; mechanand2003@gmail.com; 2 Centre for Materials Engineering and Regenerative Medicine, Bharath Institute of Higher Education and Research, Chennai 600073, India; mohanavel2k16@gmail.com; 3 Department of Mechanical Engineering, Saveetha School of Engineering, SIMATS, Chennai 602105, India; sathish.sailer@gmail.com; 4 School of Mechanical & Aerospace Engineering, Nanyang Technological University, 50 Nanyang Avenue, Singapore 639798, Singapore; amma0002@e.ntu.edu.sg; 5 Department of Mechanical and Industrial Engineering, College of Engineering, Majmaah University, Al-Majmaah, Riyadh 11952, Saudi Arabia; i.alarifi@mu.edu.sa; 6 Engineering and Applied Science Research Center, Majmaah University, Al-Majmaah, Riyadh 11952, Saudi Arabia; 7 Research Center for Advanced Materials Science (RCAMS), King Khalid University, Abha 61413, Saudi Arabia; irfan@kku.edu.sa; 8 Mechanical Engineering Department, College of Engineering, King Khalid University, Abha 61411, Saudi Arabia; aanqi@kku.edu.sa; 9 Department of Mechanical Engineering, Glocal University, Delhi-Yamunotri Marg, SH-57, Mirzapur Pole, Saharanpur 247121, India; 10 Department of Mechanical Engineering, P. A. College of Engineering (Affiliated to Visvesvaraya Technological University, Belagavi), Mangaluru 574153, India

**Keywords:** materials selection, polymer, composites, properties, Taguchi

## Abstract

Two-body abrasive wear behavior of glass fabric reinforced (GC) epoxy and titanium dioxide (TiO_2_) filled composites have been conducted out by using a tribo test machine. GC and TiO_2_ filled GC composites were produced by the hand layup technique. The mechanical performances of the fabricated composites were calculated as per ASTM standards. Three different weight percentages were mixed with the polymer to develop the mechanical and abrasive wear features of the composites. Evaluation Based on Distance from Average Solution (EDAS), a multi-criteria decision technique is applied to find the best filler content. Based on the output, 2wt% TiO_2_ filler gave the best result. Abrasive wear tests were used to compare GC and TiO_2_ filled GC composites. The abrasion wear mechanisms of the unfilled and TiO_2_ filled composites have also been studied by scanning electron microscopy. The outcome of the paper suggests the correct proportion of filler required for the resin in order to improve the wear resistance of the filled composites. Taguchi combined with Multi-Criteria Decision Method (MCDM) is used to identify the better performance of the TiO_2_ filled epoxy composites.

## 1. Introduction

Filler selection is very important in determining the important properties of a composite. Properties such as the stiffness or rigidity, tensile, abrasion resistance, and impact force of a composite are developed after the addition of fillers to the matrix. They are also called strengtheners because they hold a major role in strengthening the composites. In the field of filler filled polymer composites, there is no restriction on the usage of type of filler. Among the broad range of materials available as fillers, the relatively simple chalks and limestones to complex rare-earth magnetic powders can be used as fillers. Even, apparently, mundane and relatively inexpensive fillers can vary in subtle, but important, ways according to their precise origin and method of manufacture, thus adding their interest to the scientific and technological mind. The first step in the process of wear is the selection of the base polymer to be used. This choice will be determined principally by the end-product specification and/or service conditions. This will stipulate such conditions as upper and lower load, abrasive distance, and level of dry or wet, mechanical, etc. This task requires a comparison of the basic properties of the polymer types available with a view to make a selection on an economic basis.

Still there are certain areas where polymers alone cannot satisfy the extreme wear condition environment. To solve this reinforcement in form of fiber/filler or combination may be added to the polymer. Different researchers have analyzed the diverse fillers included with the polymers, and the characteristics of the resins were greatly improved. Iskender Ozsoy et al. [1] evaluated the effect of the large and small size filler content ratios of the mechanical characteristic of polymer composites. Micron size fillers of aluminum oxide, TiO_2_, and fly ash were put in at a percentage of ten to thirty by weight. Nano size fillers of aluminum oxide, TiO_2_, and nano clay were included by two to ten weight proportions. They examined that the mechanical characteristics of the filled composites amplified with rising large and small size filler content. Singh et al. [2] evaluated and estimated the mechanical and erosive wear features of TiO_2_ and ZnO filled woven E-glass fiber-based vinyl ester composites. They concluded that TiO_2_ filled composites were examined to achieve better success than a zinc oxide filled matrix in an erosive wear environment.

Choi et al. [3] compared the tensile strength of the nano TiO_2_, filled and unfilled, epoxy composites. The crack resistance of the TiO_2_ significantly improved the tensile strength of the nano TiO_2_ filled over that of pure epoxy. They also investigated the effect of particle size on the mechanical properties of the TiO_2_-epoxy nano composites.

Ramesh et al. [4] studied the mechanical performance of diverse micro modifiers such as Al_2_O_3_/SiO_2_/TiO_2_ with equal wt% for glass fiber/epoxy hybrid composite. They examined that alumina modified epoxy composite exhibited better mechanical characteristics compared to the other modifiers. The mechanical and thermal characteristics of glass fiber reinforced polymers were significantly improved after the accumulation of 7.5 wt% of basalt powder [5].

Sarkar et al. [6] examined tribological (sliding) wear of woven glass epoxy composites packed with Aluminum granular. Among the different wt% (5, 10, 15, and 20 wt%) of Al powder, the 5 wt% Al filled composite acquiesces less friction coefficient and better wear resistance. Fillers are added to both thermoplastic [7,8,9,10] and thermosetting [11,12,13,14,15,16] resins to progress the mechanical characteristics, as well as tribological characteristics, of the composites. Characterization of TiO_2_ filled epoxy composites under sliding wear has been executed by Siddhartha et al. [17]. In their research, an experimental approach, based on Taguchi’s parameter design, was employed to evaluate the outcome of different factors and their relations.

Pervez et al. [18] optimized the performance characteristics of nano TiO_2_ filled HDPE composites. They concluded that the percentage of nano-TiO_2_ filler was the most significant control factor by using the ANOVA Technique. For fabrications of optimum quality, the Grey relational analysis was chosen to optimize the operating conditions.

The scientific writings of the published journals examined that TiO_2_ composites have been used for wear resistance applications. As per various literature outcomes, no author endeavored the work on two-body abrasive wear of TiO_2_ filled glass fabric reinforced epoxy composites. In the current work, the interaction of parameters was investigated using the Taguchi method. Kumar et al. [19] analyzed the Taguchi scheme to establish the process parameter for the load and stress of wood dust reinforced polymers. With the assistance of the Taguchi technique, the outcome of load, abrading distance, sliding speed, and percentage of filler on the abrasive wear of the filled composite was studied [20]. Swain et al. [21] calculated the parametric analysis of abrasive wear by using the Taguchi experimental design for the three-body abrasive wear of jute fabric incorporated epoxy composites. The optimal conditions to minimize the shrinkage of talc and glass fiber reinforced polypropylene composites were obtained through ANOVA, the Taguchi method, and regression analysis. The authors identified that reinforcement type had a major influence on shrinkage compared to other factors [22].

The effects of different wear parameters on the sliding wear performance of AA6063/SiC co-continuous composite were analyzed by using Taguchi optimization method. The ANOVA technique is used to identify the most significant factor for the wear behavior of composites [23]. Different researchers analyzed the mechanical and wear characteristics of the composite, and the various process parameters and their interactions were examined by using the Taguchi method.

In the current research work, TiO_2_ was added as filler for the epoxy composites. How much amount required is the big question to be answered. If the particle size is small, a minimum weight percentage is required [24]. If the particle size is larger, a high amount of weight of percentage is added for the improvement of properties. Therefore, the optimum filler content has to be determined. To solve this, the multi-criteria decision method (MCDM) is used. This method is used to solve the problem by dividing large into smaller sections, and the researchers analyze it to sort out the topics with sensible result. MCDM is reliable, very easy to pertain, and has several techniques. Material scientists use these techniques to work out the problems of options of site, supply chain, and selection of materials for the concept of mechanical network [25]. Keshavarz et al. [26] showed that the EDAS method is more competent than the Technique for Order of Preference by Similarity to Ideal Solution (TOPSIS) method with respect to the defined rank reversal measure.

Kumar et al. [27] develops an MCDM hybrid approach for selecting the best femoral component of total knee replacement. The final rank of the selection of material was achieved by combining the five MCDM methods. Among the five different methods, EDAS was one of the methods used to find the rank of materials for total knee replacement.

On account of the above: the current work contracts with the optimization of filler content on the two-body abrasive wear of the TiO_2_ filled glass-fiber reinforced polymer composites by using the Taguchi technique. EDAS is used to determine the best filler content to progress the mechanical and abrasive characteristics of the filler filled composites

There are various MCDM methods available to determine the best option from the list of materials. The unique method of EDAS is the rank reversal measure over the other methods. In order to identify the best filler content, EDAS, a MCDM technique, is used for TiO_2_ filler filled epoxy composites. From the literature review, it is understood that the optimum filler ratio for TiO_2_ filled epoxy composites, and the combined Taguchi and EDAS, is not applied for filler filled composites. In this regard, the current work focuses on the investigation of the effects of different factors like load, speed, and distance on the abrasive wear behavior of TiO_2_ filled glass fiber reinforced epoxy composites. The objective is to have a more significant factor of their performance on the above-mentioned parameters by using Taguchi technique. The effect of the addition of TiO_2_ on the mechanical properties (like tensile, compression, impact, and inter laminar shear strength) was also studied.

## 2. Materials

**Figure 1:**
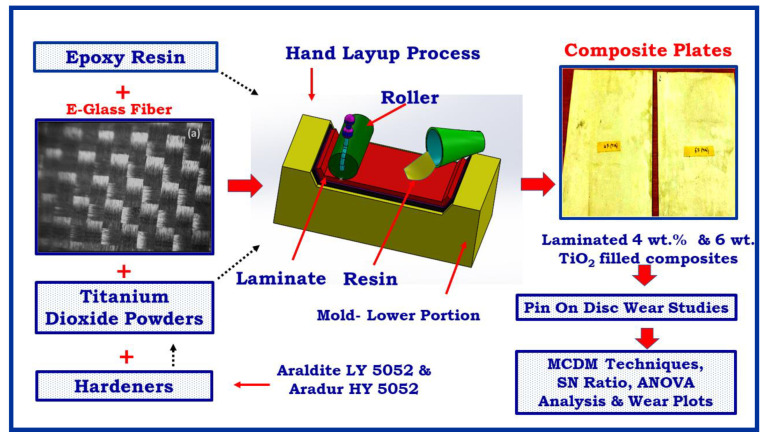
Micro-structural image for E-glass fiber, photographic image of laminated 4 wt% and 6 wt. TiO_2_ filled composites and process flow of present work.

E-Glass fiber of architecture bidirectional fabric 360 gsm was acquired from M/s. Sakthi fibres, Chennai, India. A glass fiber with a diameter of 18 μm was utilized as the strengthening agent, which is exposed in Figure 1. Araldite LY 5052 and aradur HY 5052 were procured from M/s. Huntsman Advanced Materials, Mumbai, India. The LY 5052 is a low viscosity liquid of 1000–1500 cP and density is 1.17 g/cc. The hardener HY 5052 having viscosity of 40–60 cP and density is 0.94 g/cc. TiO_2_ powder of 10 μm size is purchased from Sigma-Aldrich, Chennai, India.

### 2.1. Fabrication of Composites

The composites have been fabricated in laminates of 2.5 mm thick using hand layup technique. The manufacturing process of hand lay-up is as follows: Before adding fiber and filler to the resin, hardener and resin has to be cured. For curing, as per the supplier suggestion, the hardener to epoxy ratio was taken as 38:100. Precut of reinforcing layer is set on the wet resin. Air is taken out by wiping the brush. The reinforced glass fabric layers are immersed in the resin. Consequently, additional layers assemble in order to get the laminate thickness of 2.5 ± 0.3 mm. After that, the laminate was cured in the autoclave at a temperature of 80 °C for 24 h. Finally, the laminated plates are removed and trimmed to the required size. To prepare the TiO_2_ filled glass epoxy composite, a 2 wt% is included in the epoxy mixture and mixed well. The plate of dimension 200 mm × 200 mm × 2.8 + 0.2 mm (Figure 1) was constructed. Samples for the necessitated size were prepared by means of a cutter. Table 1 presents the details of the fabricated specimens.

### 2.2. Techniques

**Figure 2:**
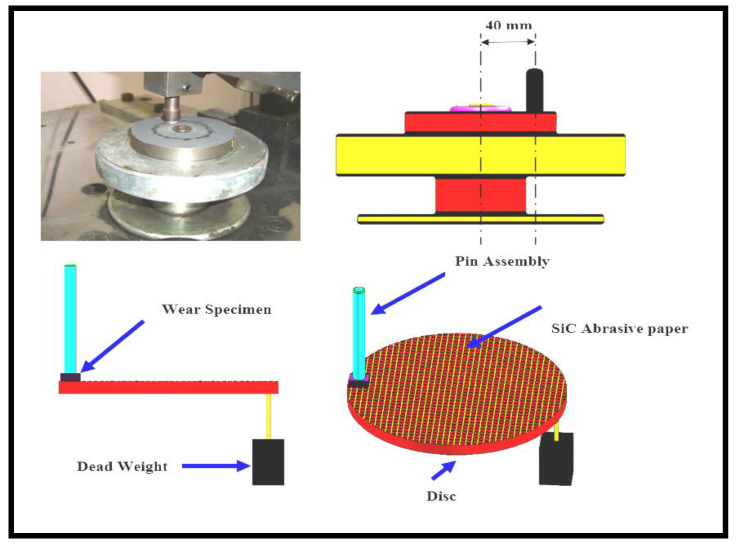
Schematic illustrations of a pin disc machine and image of a two-body wear abrasive set up.

The two-body abrasive wear performance of the fabricated composites was tested by using a pin-on-disc machine as per ASTM-G99 standard. The required specimen of 5 mm × 5 mm × 2.8 mm pasted to a pin having cylindrical shape. The pin is having Ø10 mm and 25 mm height, which is mentioned in Figure 2. To make abrasive contact, the pin makes contact with the SiC abrasive paper coated disc. Once the test completed, the abraded SiC paper was removed. For the next test new SiC paper was stuck to the disc. The pin having specimen was located on a path of Ø 20 mm in the disc. The details of the machine and sample are illustrated in Figure 2. The test was conducted by changing a different load of 5, 10, and 15 N at 200 rpm. For all the tests, abrading distances of, 25, 50 and 75 m were chosen to determine the wear resistance under multi-pass conditions. To remove the dust and unwanted particles from specimen, it is cleaned with acetone on the surface and dried for a few minutes. To determine the wear loss, initial and final weights are noted by means of an electronic weighing balance. Average values of the tested specimens are mentioned in the results. All the mechanical tests of the composites were conducted as per the ASTM standards. Figure 2 indicates the image of two body abrasive wear set up.

### 2.3. MCDM Technique

Among the three different weights% of TiO_2_ added to the resin, the best filler content is identified by the EDAS method. The procedure for to find the filler content is given below:

#### Evaluation Based on Distance from Average Solution (EDAS)

Steps involved in determine the ranking of materials is given by the following 7 steps;

Step-1 the average solution is calculated by using the following equation (*AV_j_)*

(1) $$AV_{j}\frac{\sum_{i=1}^{n}x_{ij}}{n}$$

*X_ij_* is the element of the decision matrix for *i*th alternative in *j*th attribute
*ω_j_* is the weight of attributes
*n* is the number of attributes

Step 2 the positive distance from average (PDA) is computed by non-beneficial criterion.

If *j*th principle is non-beneficial,

$$PDA_{ij}=\frac{\max(0,{\left(x_{ij}-AV_{j})\right)}_{}}{AV_{j}}$$

If *j*th principle is beneficial

(2) $$PDA_{ij}=\frac{\max(0,(AV_{j}-x_{ij}))}{AV_{j}}$$

Step 3 the negative distance from average (NDA) is determined by Equation (3)

If *j*th principle is valuable

$$NDA_{ij}=\frac{\max(0,(AV_{j}-x_{ij}))}{AV_{j}}$$

If *j*th decisive factor is non-beneficial,

(3) $$NDA_{ij}=\frac{\max(0,(x_{ij}-AV_{j}))}{AV_{j}}$$

Step 4 calculate the weighted sum of PDA

(4) $$SP_{i}=\sum_{j=1}^{m}\omega_{j}PDA_{ij}$$

Step 5 the weighted sum of NDA is evaluated by using Equation (5)

(5) $$SN_{i}=\sum_{j=1}^{m}\omega_{j}NDA_{ij}$$

Step 6 normalize the assessments of SP and SN

(6) $$NSN_{i}=1-\frac{SN_{i}}{\max_{i}(SN_{i})}$$

(7) $$NSP_{i}=1-\frac{SP_{i}}{\max_{i}(SP_{i})}$$

Step 7 standardize the values of NSP and NSN

(8) $$AS_{i}=\frac{1}{2}(NSP_{i}+NSN_{i})$$

The values of the weights are 0.25.

### 2.4. Taguchi Method

In this work, L_9_ is adequate to conduct the experiments. This array assigned by the symbol L_9_ is utilized to design experiments up to 4 independent factors. This array has 9 rows and 4 columns. Hence, an overall of 27 experiments required to enhance the factors. In the case of the Taguchi technique, it proposes that only 9 experiments are adequate to enhance the factors. Table 2 indicates the OA with coded units. The array obliges all experiments to design and run all factors as identical experiments. Designers choose diverse descriptions for the columns, but the nine trial runs will interact with all factors which is independent of column definition. OA is selected in such a way that the required experiments are chosen as per the level combinations. Table 2 also indicates the OA with uncoded units. It is important to assess the output under the most favorable situations. Signal to ratio analysis concludes the stout set of operating conditions.

## 3. Results and Discussion

The density of the composite after fabrication is measured by using the Archimedes principle (buoyancy method). Archimedes principle states that a body immersed in a fluid apparently loses weight by an amount equal to the weight of the fluid it displaces. This method allows determination of the density of solids. The weight of the composites was measured in air and then in water. The density of the composite is calculated by using the following expression.

(9) $$\rho =\frac{A}{A-B}\left(\rho_{o}-\rho_{L}\right)+\rho_{L}$$

where,

*ρ* = density of composites
A = weight of composites in air
B = weight of composites in water
$\rho_{o}$ = density of water (0.99567 g/cm^3^)
*ρ*_L_ = density of liquid displaced

It may be noted that the composite density values, calculated theoretically from weight fractions using Equation (8), are not in agreement with the experimentally determined values. Theoretical densities were calculated by Equation (9)

(10) $$\rho_{ct}=\frac{1}{\frac{w_{f}}{\rho_{f}}+\frac{w_{m}}{\rho_{m}}}$$

where, *w* and *ρ* represent the weight fraction and density, respectively. The suffix *f*, *m* and ct stand for the fiber, matrix, and the composite materials, respectively. The fabric content of composite samples is determined by a resin burn-off test according to the ASTM D3 171 specifications. The burn-off experiments were done for the composites to determine the mass content of resin and fiber. The wt% fabric (x) is determined from the following Equation (10), where, *w* and *ρ* represent the weight fraction and density, respectively. The suffix *f*, *m* and ct stand for the fiber, matrix, and the composite materials, respectively. The fabric content of composite samples is determined by a resin burn-off test according to the ASTM D3 171 specifications. The burn-off experiments were done for the composites to determine the mass content of resin and fiber. The wt% fabric (x) is determined from the following Equation (10),

(11) $$x=\frac{w}{w_{0}}\times 100$$

where w_0_ and w are the initial weight and weight after the matrix burn-off, respectively. Void content is calculated from the difference between theoretical density and actual density.

The theoretical and measured densities of all composite samples, along with the corresponding weight fraction of voids, are presented in Table 3.

It is clear from Table 3 that the 3.59% of voids in unfilled G-E composites is small compared to the filled one, this may be due to the absence of filler. With the addition of filler content more voids are found in the composites and with 2, 4, & 6 wt% of TiO_2_ as filler. The fraction of voids is also found to be increasing by increasing the weight percentage of filler. Generally, the density of a composite depends on the relative proportion of matrix filler and reinforcing materials. In the present work, an increase in filler content leads to an increase in density for the composites.

The void content is the cause for the difference between the values of actual density and the theoretically calculated one. The voids significantly affect some of the mechanical properties and even the performance of composites in the place of use. The knowledge of void content is desirable for estimation of the quality of the composites. It is understandable that a good composite should have fewer voids. However, presence of void is unavoidable in composite making particularly through hand-lay-up route.

The mechanical test confirmed that the incorporation of TiO_2_ powder in glass fiber reinforced polymer has exposed hopeful results. All the mechanical characteristics tested were tabulated in Table 4. Table 4 provides the data of the mechanical test results of the tested specimens. The tensile test was carried as per ASTM D 3039. Compression tests of the filled and unfilled composites were done as per ASTM D 695. The interlaminar shear strength test was conducted as per ASTM D2344-84 and span to depth ratio for specimen was at 5:1. For an impact test, the specimen measuring 55 mm × 12.7 mm × 2.5 mm was cut by the diamond tipped cutter. All the impact tests were conducted as per ASTM D 256. For the compression strength, there is an increase in value after the 2 wt% addition of TiO_2_. This may be due to the combined action of filler and powder, which leads to the better load bearing capability of the composites during compression. Because of stress, due to compression, it performs closing cracks and defects formed in the composites [28]. Higher amount of filler is not able to provide the closing crack mechanism due to lack of space to occupy. Hence, GC6 with 6 wt% TiO_2_ particle content has less value compared to GC4 and GC2. This may also be owing to unequal distribution of powder at elevated filler amount. Uniform distribution did not simply occur for GC6 & GC4 composites due to the reduced inter particle distance [29].

The results of calculations carried out according to the proposed methodology, based on the EDAS method, are summarized in Table 5, Table 6, Table 7, Table 8 and Table 9. The positive distance from average were calculated and mentioned in Table 5. The weighted sum of PDA is calculated as per the procedure and the values are presented in Table 6. Similarly, Negative Distance from Average and weighted are calculated and mentioned in Table 7 and Table 8. The average solution of each characteristic is attained and presented in Table 5. According to the credence of attributes, the weighted positive distances from average solutions are concluded in Table 6. Table 7 indicates the other values of the negative distance from average solution. Based on the appraisal score of each alternative AS_i_ value, the ranking order was determined, and a “GC2” was recognized as the best alternative to enhance wear resistance for TiO_2_ filled epoxy composites, and the alternatives are ranked as follows: GC > GC6 > GC4.

The EDAS method is very practical in conditions with the contradictory attributes, and the best alternative is chosen by calculating the distance of each alternative from the optimal value. The EDAS method is applied in the evaluation of airline services, solving air traffic problems and personnel selection.

### 3.1. Analysis of Control Factors

Measure of confidence is analyzed by using an analysis of variance technique called ANOVA. By this method, the data cannot be directly analyzed but used to determine the variance of the data. The L_9_ orthogonal array is used to govern the outcome of a variety of control factors. By using Minitab software, data analysis was executed. Table 10 and Table 11 indicates the response table for S/N ratio and means, respectively.

**Figure 3:**
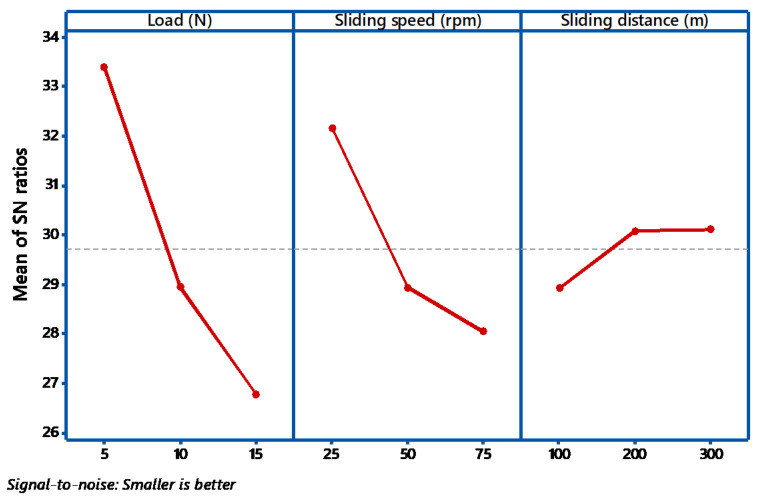
Main effect plot (S/N ratio).

**Figure 4:**
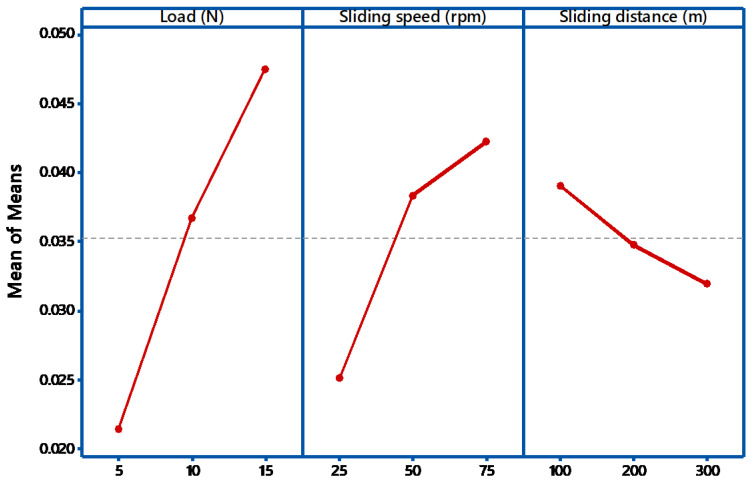
Main effect plot (Mean).

The S/N ratio on the wear loss during abrasive wear is exposed in Figure 3. It shows how the Signal to Noise ratios are employed to calculate the main effects, in addition to the estimated performance, at the most favorable state. During the abrasive wear test, if there is a little alteration in the parameter, then it has a direct control on the wear behavior. All the three factors have minimum value say 25 for load and 75 for speed. If the observed graph is horizontal, then the factor is less significant. In this case from Figure 3 and Figure 4, all the graphs are not nearly horizontal. In the main effects plot for S/N ratios, the mean of S/N ratios for each factor category is plotted against the test level for each factor. A horizontal dotted line is drawn for all the graphs of the main effect. From ANOVA analysis, if the angle of inclination is more, with respect to the reference, then that factor has considered to be most significant [30]. From the Figure 3, it is examined that load factor has the maximum inclination compared to other factors. Similarly, if there is less inclination, then it has very slight significance, so in our case, the sliding speed has slight significance. If the curve is almost parallel to the reference line, then it indicates no significance. From the plot, it is clearly identified that sliding speed has no noteworthy effect on the weight loss of the composites. From the main effects plot, it is perceived that applied load has a significant influence on wear, followed by sliding speed and sliding distance. Similar results were obtained by Nayak et al. [31]. In evaluating the result at the most favorable circumstance, the factors responsible for the significant contributions are incorporated. From the results obtained, load dominates the rank 1, followed by sliding speed and sliding distance.

Table 12 illustrated the o/p of the ANOVA with the weight loss of TiO_2_ filled composites of parameters such as load, sliding speed, and sliding distance. NOVA results are framed to split up the individual effects from all of the parameters. The percentage role of each parameter is computed and tabulated in Table 12. The order of significant parameters is also calculated and presented in the last column of Table 12. Percentage contributions offer an enhanced signal of the control of individual factors. With the results obtained from Table 12, the ANOVA results in relation to weight loss show that load has the highest significant contribution of 61.6% with the *p*-value of 0.074, pursued by sliding speed with 28.8% contribution and the *p*-value of 0.145. Significance level of the parameters is also checked with the help of the *p*-value. Basically, the value of P lies between 0 and 1. If the obtained *p* value is small, then the parameter has a statistically significant output on the weight loss of the composites. It can be calculated as the ratio of Adj_SS_ to the total value of Adj_SS_.

Table 12 presented the model summary of the parameters with R-sq as a regression coefficient. Regression coefficient indicates that the output data is close in order to fit the regression line. The calculated R-sq value is 95.09% which represents that the data obtained are significant. The value of R-sq 0.8035 provides an excellent fit to the data. The value S represents the standard deviation of all the data points and relates the data and the fitted value points. If the obtained S is significantly less, it means the model describes the output in a better way.

The regression equation of wear loss (W) in g is conveyed in term of the un-coded values of the independent parameters in the Equation (12) given below:

Wear loss (g) = 0.03526–0.01376 Load (N)_5 + 0.00151 Load (N)_10 + 0.01224 Load (N)_15–0.01009 Sliding speed (rpm)_25 + 0.00311 Sliding speed (rpm)_50 + 0.00698 Sliding speed (rpm)_75 + 0.00378 Sliding distance (m)_100–0.00049 Sliding distance (m)_200–0.00329 Sliding distance (m)_300 (12).(12)

This fitting equation is dependent on the wear parameters such as distance, sliding speed and load.

The wear loss is presented here to identify the best component for the abrasive wear environment. Minimum wear loss gives the good abrasive wear material. The role of this equation gives the major parameter that influences the wear loss of the tested specimens.

**Figure 5:**
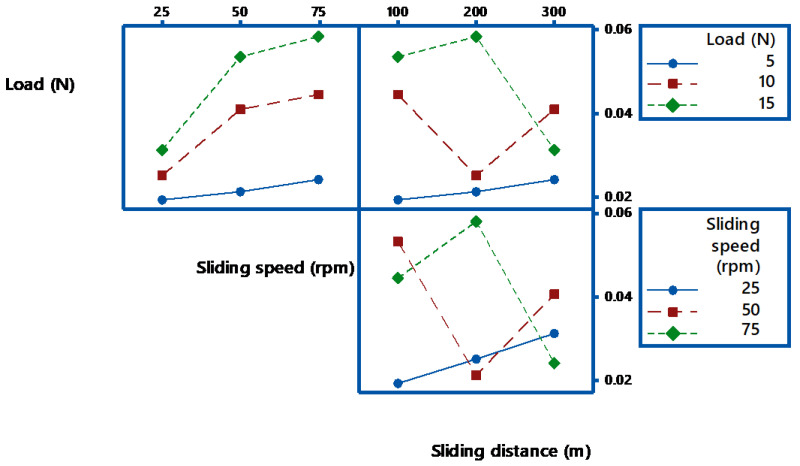
Interaction plot for wear loss.

The interaction plots for wear loss are analyzed based on the non-parallelism of the parameter effects. Figure 5 shows the interaction plot for wear loss. Generally, the lines of interaction of plots are not parallel there is a strong interaction between the factors. Contradict to this; there is a nominal interaction if they are parallel. From the interaction plots of Figure 3, the lines are parallel indicates that there is a strong interaction between the applied load and sliding distance. It is observed that applied load (L) is the most influencing parameter for the wear characteristics of filled composites.

### 3.2. Influence of Process Parameters

**Figure 6:**
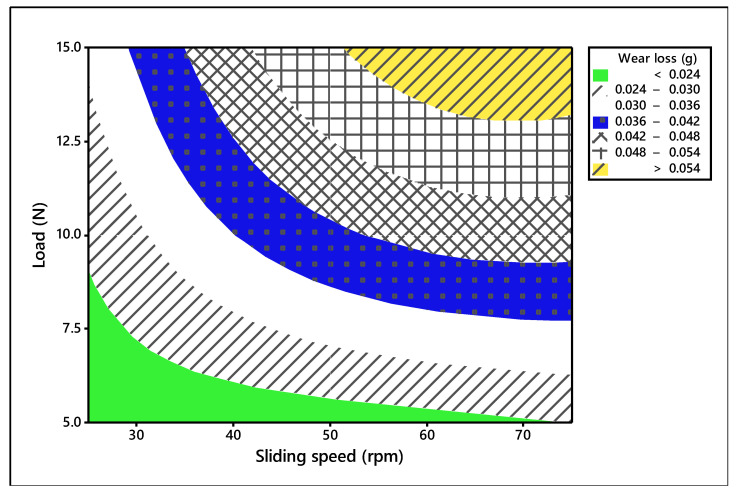
Contour plot for wear loss (Load vs. sliding speed).

**Figure 7:**
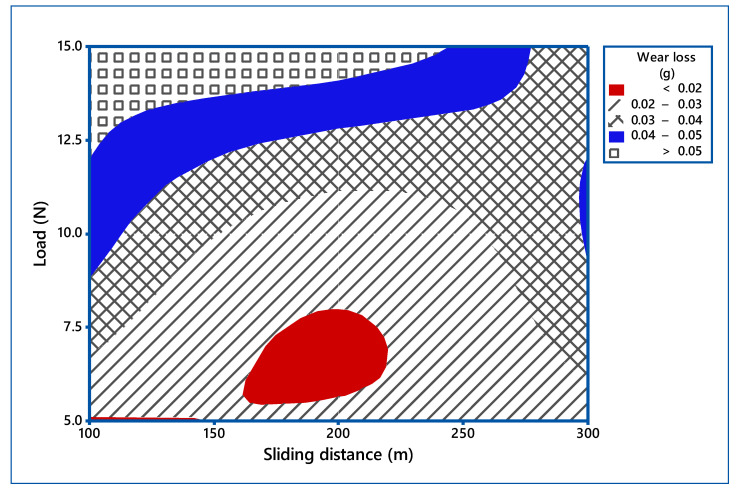
Contour plot for wear loss (Load vs. sliding distance).

**Figure 8:**
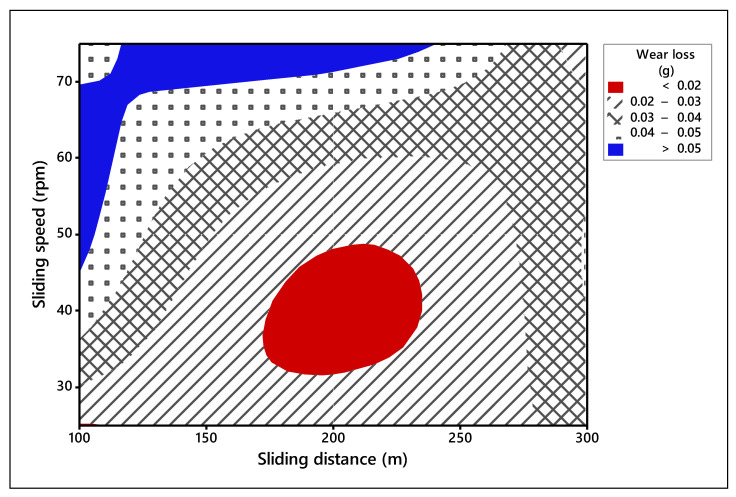
Contour plot for wear loss (Sliding speed vs. sliding distance).

**Figure 9:**
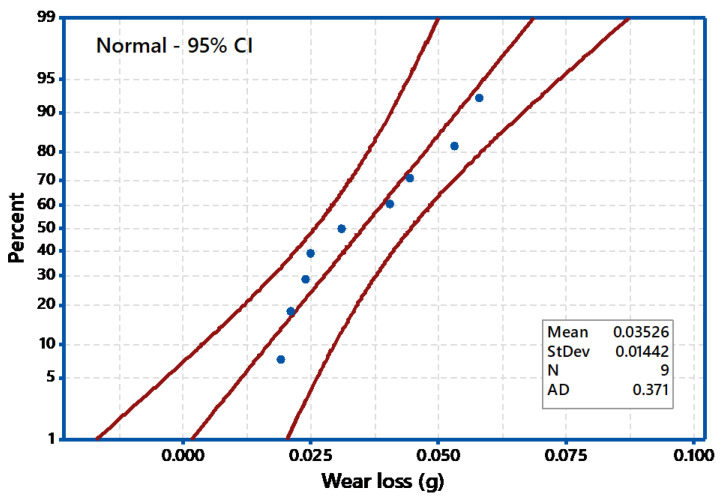
Probability plot for wear loss.

The process parameters which influence the wear behavior on the TiO_2_ filled GE composite have been evaluated using 3-D surface plots and 2-D Contour plots. The contour plots are based on three levels and two parameters in the *X* and *Y*-axis, as shown in Figure 6, Figure 7 and Figure 8, and the shaded region shows the response of the wear. Contour plots depict wear regions, clearly based on interactions between abrasive wear test process parameters. It is noted from Figure 6 that sliding speed and applied load interaction contour plot signify that load applied less in quantity and reduced sliding speed produce less wear loss over abrasive surfaces. It is observed that whenever applied load is increased in wear test, it decreases the sliding speed of the disc. When the parameters increase from low level to high level, the wear of the composites is also enhanced under all test conditions. Additionally, other interaction effects of various parameters on wear are revealed in contour plots 8 and 9. Contour plots of wear confirm the same effect shown in ANOVA table. Hence, it is concluded from contour plots that interaction effect between applied loads and sliding speed dominates the wear loss of the abraded surfaces. The surface plot also indicated the same. The Figure 9 depicts the probability plot for wear loss of the TiO_2_ filled composite.

### 3.3. Worn Surface Morphology

**Figure 10:**
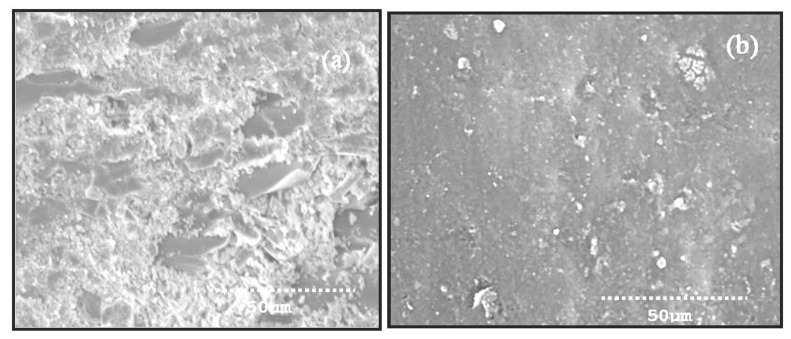
SEM of worn surface of (**a**) GC (**b**) GC 2 composite at 15N load, sliding distance of 75 m.

The SEM of the abraded composites was observed by using a modelS3000, V-1, HITACHI. The morphology characteristics of the abraded planes of GC and GC2 were shown in Figure 10a,b. Figure 10a signified that the exposure of fibers were observed to be more. Because of the sharp silicon, carbide particles easily penetrated into the weak resin surface and removed it during the abrasion process. The Figure 10a displayed resin fracture, wear debris of broken fibers at all layers. This is due to that, at higher abrading distance, the combination of load and more contact time lead to pulverization of fibers. During abrasion of high loads, the contact time between the load and the fiber were increased which leads to the pulverization of fibers. Analogous consequences, such as deep furrows because of matrix removal, were acquired by Senthil et al. [32]. The depth of furrows depends upon the ploughing action of sharp SiC abrasive particles in the abrasion condition. The failure mechanism of the GC composites takes place in the following manner: initially, fiber starts failure with micro-cracking and next, the breaking of fibers occurs, and finally, removal of fiber takes place. From Figure 10a, a greater number of inclined fractures of fibers, and less attachment between fiber and resin, are visible on the worn surface of the GC composites. In general, surface boundary of GC composites signified additional fiber pulverization, and furthermore inclined breakage of fiber and less fiber matrix de-bonding [33,34,35,36,37,38,39].

Figure 10b represents the worn surface of the GC2 composites at higher abrading distance. The amount of TiO_2_ acts as a warrior to prevent damage to fiber and matrix during loading conditions. It is also evidenced from the Figure 10b. The inclined fracture and matrix removal is less in GC2 composites as compared to GC composite under abrasive wear circumstances. In this case, there are considerable contacts between glass fiber and TiO_2_ filled matrix, which has effects in good bonding. This effect is in accord with the SEM image (Figure 10b). Additionally, matrix wear, inclined fiber fracture, and furrows are not noticed in GC2 composite. The wear mechanism of the GC2 composite indicates that, even during heavy loading and high abrading distance, a superior conjoin link is maintained between TiO_2_ powder and resin. The fiber fracture and matrix damage is comparatively nothing, considering with the GC composite. The reason may be due to the presence of TiO_2_ powder in the matrix, which strongly opposes the infiltration of SiC pieces during abrasion.

## 4. Conclusions & Observations

EDAS technique identified that among the three different weight percentages, 2 wt% TiO_2_ filled epoxy composite gives the best filler content under all mechanical testing conditions. This technique is regarded as one of the novel systems in MCDM, which has been examined in a little time. The target-based attributes related mechanical properties such as tensile strength, compression strength, inter-laminar shear strength, and impact strength of the composite materials are considered in this study. This system assesses diverse attributes in the existence of conflicting parameters. Additionally, the best choice is identified by resolving the optimistic and pessimistic distances from the most favorable quantity. The most favorable value is calculated from the normal assessments of decision matrix. 

The Taguchi method analyzed the interaction of the parameters as well as the regression equation of the wear loss. From the ANOVA results, it is observed that the most influential factors in terms of percentage contribution affecting the abrasive wear of TiO_2_ filled epoxy composites are load (61.6%), sliding speed (28.8%), and sliding distance (4.57%). The wear loss of the GC2 composite decreased with the inclusion of 2 wt% TiO_2_ content. SEM studies of the worn surfaces revealed that the wear technique for the GC composites was more inclined fiber breakage and matrix wear. The wear mechanism in GC2 composites has no matrix wear and fiber exposure.

## Figures and Tables

**Table 1:** Detail of the fabricated specimens.

Sl. No.	Composite	Fibre (wt%)	Resin (wt%)	Filler (wt%)
1	GC	60	40	0
2	GC2	60	38	2
3	GC4	60	36	4
4	GC6	60	34	6

**Table 2:** L_9_ OA with coded units and L_9_ orthogonal array with un-coded units.

Experiment Number	Load (N)	Speed (rpm)	Distance (m)	Load (N)	Speed (rpm)	Distance (m)
1	1	1	1	5	100	25
2	1	2	2	5	200	50
3	1	3	3	5	300	75
4	2	1	2	10	100	50
5	2	2	3	10	200	75
6	2	3	1	10	300	25
7	3	1	3	15	100	75
8	3	2	1	15	200	25
9	3	3	2	15	300	50

**Table 3:** Theoretical and measured densities along with void fractions in composites.

Sl. No.	Composite	Theoretical Density (g/cm^3^)	Actual Density (g/cm^3^)	Void Fraction (%)
1	GC	1.67	1.611	3.59
2	GC2	1.726	1.660	3.82
3	GC4	1.781	1.705	4.26
4	GC6	1.839	1.745	5.11

**Table 4:** Mechanical properties of filled composites.

Sl. No.	Material	Tensile Strength (TS) (MPa)	Compressive Strength (CS) (MPa)	Interlaminar Shear Strength (ILSS)	Impact Strength (IS) (J)
1	GC	133.43	166.18	3.25	5.3
2	GC2	145.74	182.17	4.225	8.72
3	GC4	157.05	138.61	1.197	12.07
4	GC6	175.74	126.6	0.501	18.17

**Table 5:** Positive Distance from Average (PDA).

Sl. No.	Material	TS	CS	ILSS	IS
1	GC	0	0.083382	0.417203	0
2	GC2	0	0.187626	0.842363	0
3	GC4	0.026538	0	0	0.090826932
4	GC6	0.148703	0	0	0.642114776

**Table 6:** Weighted sum of PDA.

Sl. No.	Material	TS	CS	ILSS	IS	Sp_i_
1	GC	0	0.020846	0.104301	0	0.125146
2	GC2	0	0.046907	0.210591	0	0.257497
3	GC4	0.006634	0	0	0.022707	0.029341
4	GC6	0.037176	0	0	0.160529	0.197704

**Table 7:** Negative Distance from Average (NDA).

Sl. No.	Material	TS	CS	ILSS	IS
1	GC	0.127851	0	0	0.521012201
2	GC2	0.047389	0	0	0.211929507
3	GC4	0.026538	0.096356	0.478033	0
4	GC6	0.148703	0.174653	0.781533	0

**Table 8:** Weighted sum of NDA.

Sl. No.	Material	TS	CS	ILSS	IS	SN_i_
1	GC	0.031963	0	0	0.130253	0.162216
2	GC2	0.011847	0	0	0.052982	0.06483
3	GC4	0.006634	0.024089	0.119508	0	0.150232
4	GC6	0.037176	0.043663	0.195383	0	0.276222

**Table 9:** Ranking by using EDAS.

Sl. No.	Material	SPi	SNi	NSPi	NSNi	ASi	Rank
1	GC	0.125146	0.162216	0.48601	0.412733513	0.449372	2
2	GC2	0.257497	0.06483	1.000002	0.765299084	0.88265	1
3	GC4	0.029341	0.150232	0.113948	0.456119774	0.285034	4
4	GC6	0.197704	0.276222	0.767793	−1.21686 × 10^−7^	0.383896	3

**Table 10:** Response Table for Signal to Noise Ratios (Smaller is better).

Level	Load (N)	Sliding Speed (rpm)	Sliding Distance(m)
1	33.39	32.15	28.93
2	28.95	28.93	30.08
3	26.78	28.04	30.10
Delta	6.61	4.11	1.18
Rank	1	2	3

**Table 11:** Response Table for Means.

Level	Load (N)	Sliding Speed (rpm)	Sliding Distance(m)
1	0.02150	0.02517	0.03903
2	0.03677	0.03837	0.03477
3	0.04750	0.04223	0.03197
delta	0.02600	0.01707	0.00707
Rank	1	2	3

**Table 12:** Analysis of Variance.

Source	DF	Adj SS	Adj MS	F-Value	*p*-Value	% of Contribution
Load(N)	2	0.001024	0.000512	12.54	0.074	61.6
Sliding speed(rpm)	2	0.000480	0.000240	5.88	0.145	28.8
Sliding distance(m)	2	0.000076	0.000038	0.93	0.518	4.57
Error	2	0.000082	0.000041			5.03
Total	8	0.001662				100

S: 0.0063899, R-sq: 95.09%, R-sq(adj): 80.35%, R-sq(pred): 0.52%.

## Data Availability

Data Sharing is not applicable.
